# A laparoscopic study investigating 3D vs 2D imaging systems using a pelvitrainer model with experts, non-experts, and students

**DOI:** 10.1186/s12893-020-00892-8

**Published:** 2020-11-09

**Authors:** Tibor Andrea Zwimpfer, Dominik Lacher, Bernhard Fellmann-Fischer, Michael Mueller

**Affiliations:** 1 University Hospital Basel, Gynecological Clinic, Basel, Switzerland; 2 Hospital Limmattal, Zürich, Switzerland; 3 University Hospital Berne, Gynecological Clinic, Berne, Switzerland

**Keywords:** 3D laparoscopy, 2D laparoscopy, Pelvitrainer, Standardised tasks

## Abstract

**Background:**

Vision is an essential element of laparoscopic surgery that defines the outcome of an operation in regards to time, mistakes and precision. A 3-dimensional (3D) perspective may improve vision during an operation. Therefore, this study was designed to compare 3D versus 2-dimensional (2D) perspectives using a pelvitrainer model.

**Methods:**

Fifty candidates were divided into 3 categories based on different experience levels. The candidates were randomised into two groups, with each group performing the same 4 standardised tasks. Group A approached the tasks first with 3D high definition and in a second turn with 2D high definition. Group B carried out the tasks with the systems in reverse order. Task completion time and the number of mistakes made for each task were recorded. After completing the tasks, participants answered questions concerning the two systems.

**Results:**

Group A was, on average, 20% faster at all four tasks and made approximately 18% fewer mistakes in two of the tasks in comparison to group B. The experts significantly benefited from the 3D system in terms of accuracy compared to non-experts and students. The students demonstrated a significantly greater benefit from the 3D system when performing non-linear, continuous movements. Loss of concentration occurred at the same rate for subjects using the 2D and 3D systems. Nausea and dizziness were reported only when working with the 3D system. 91% found the 3D system advantageous for accomplishing the tasks.

**Conclusions:**

Irrespective of experience level, 3D laparoscopy shows advantages in saving time, increasing accuracy and reducing mistakes. These benefits were also accompanied by subjective advantages that were noted by the participants. However, the more complex the task, the less significant the benefit of the 3D system and some people feel handicapped by the eyewear.

## Background

Laparoscopic surgery is a common procedure that offers numerous advantages including a reduction in post-operative infection and blood loss, better cosmetic outcomes and shorter hospital stays [1–3]. However, there are some disadvantages in comparison to laparotomies. For example, surgeons experience fatigue more quickly, there are only four degrees of freedom and operations take longer [4, 5]. With the help of 3-dimensional (3D) visualizations it is possible to shorten the operation time and achieve cost reductions [6]. Additionally, higher precision and fewer mistakes are made [7–9]. To date, there have been a number of other studies investigating the advantages and disadvantages of using a 3D imaging system for conventional laparoscopy (LSC) [7, 10–12]. Various study designs have been used, and most compare two different groups using either a 3D or 2-dimensional (2D) system for exercises on a pelvitrainer, or in vivo [9–12]. In our study, an objective comparison of 3D vs 2D LSC was carried out using a pelvitrainer with a variety of standardised exercises performed by laparoscopic experts, non-experts and medical students. The exercises tested surgical competence regarding time, precision and mistakes. In addition, a subjective evaluation in the form of a questionnaire was used to assess the systems. Our study hypothesis tests the idea that the application of the 3D system reduces operation time and mistakes, and optimises precision.

## Methods

### Study population

In total, 60 participants were tested and 50 measurements were used. The first 4 participants were not included because the study design was changed to incorporate a dynamic rather than static camera movement. Two of the study participants were stereo-blind according to the Lang stereo test and therefore excluded. Four candidates aborted their trials prematurely as they were interrupted due to emergency operations.

The used measurements were from fifteen experts with an average age of 48.7 years (who had conducted more than 50 laparoscopic operations per year for at least 5 years) recruited at the gynaecological laparoscopic course in Davos, Switzerland. Additionally, we selected 15 non-experts with an average age of 34.7 years (who had performed more than 10 but less than 50 laparoscopic operations), who were assistant physicians at the University of Berne and 20 medical students with an average age of 24.9 years (no experience in LSC) from the University of Berne. All participants had to give their consent to participate in this study and waive any claims. The anonymisation of personal data was guaranteed. The project is not defined as a research project according to Human Research Act Art. 2; therefore, an IRB approval and written consent is not needed.

### Study design

**Fig. 1:**
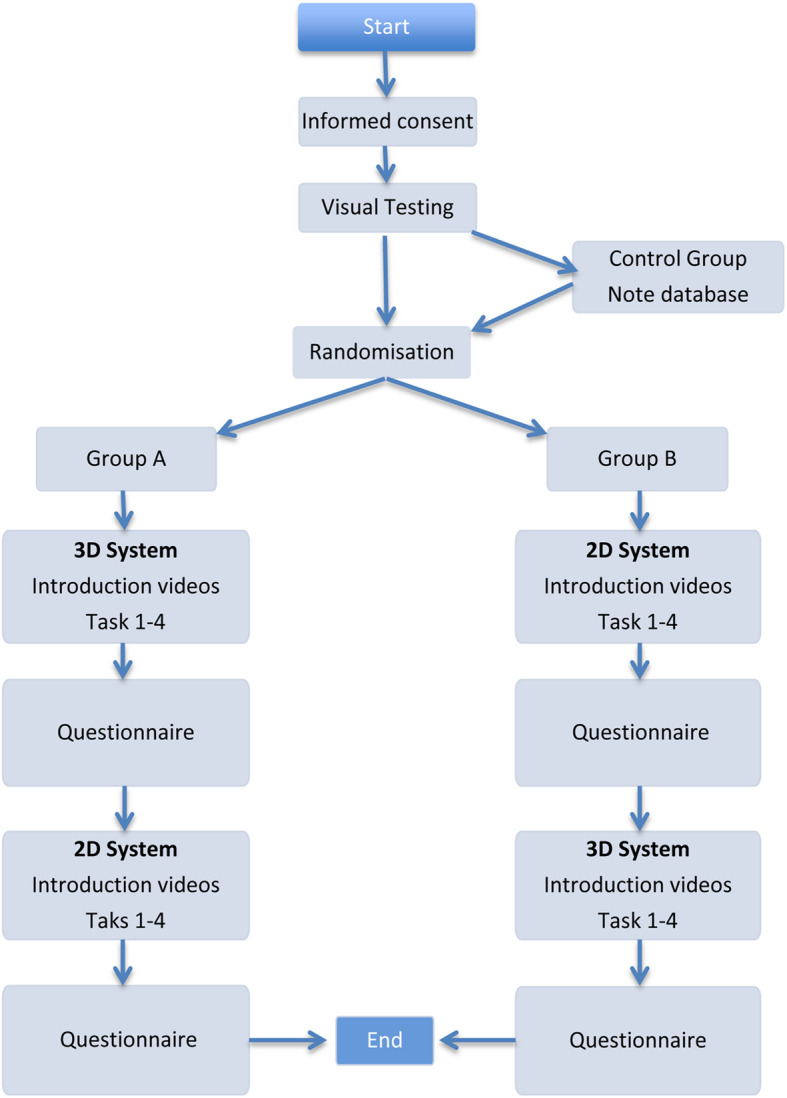
Flow chart: Measurement procedure

All participants performed four standardised exercises. They were randomly assigned to one of two groups. The group composition was structured as follows: Group A includes 11 students, 11 non-experts and 6 experts. Group B includes 9 students, 4 non experts and 9 experts. Group A first performed the standardised exercises with the 3D system and then with 2D system. Group B carried out the exercises in the reverse systems order Fig. 1. The systems used were 3D HD (high definition) and 2D HD systems by Karl Storz (Karl Storz SE & Co., Tuttlingen, Germany). Time, precision and mistakes were recorded during each subject’s performance. Once participants had completed all of the exercises they were given a questionnaire that asked about their experience with both systems.

### Visual test

The inclusion of stereo-blind people would have distorted the main study results. Therefore, participants with stereo-blindness who took part in the study were marked as a reference group and are to be evaluated separately in the analysis. Verification of stereo-blindness was accomplished using the Lang stereo test. Because of the small number in this reference group (2 study participants), a separate statistical analysis using this group was not performed.

### Instrument set-up

All exercises were carried out on two pelvitrainers constructed such that they correlated with the area surrounding the arcuate line of Douglas. Two endoscopy towers were identically set up, and both systems were equipped with a 24-in. monitor and a 300 W Xenon light source (Karl Storz SE & Co., Tuttlingen, Germany). The camera control unit possessed a capacity for videos with a resolution up to 720p.

**Fig. 2:**
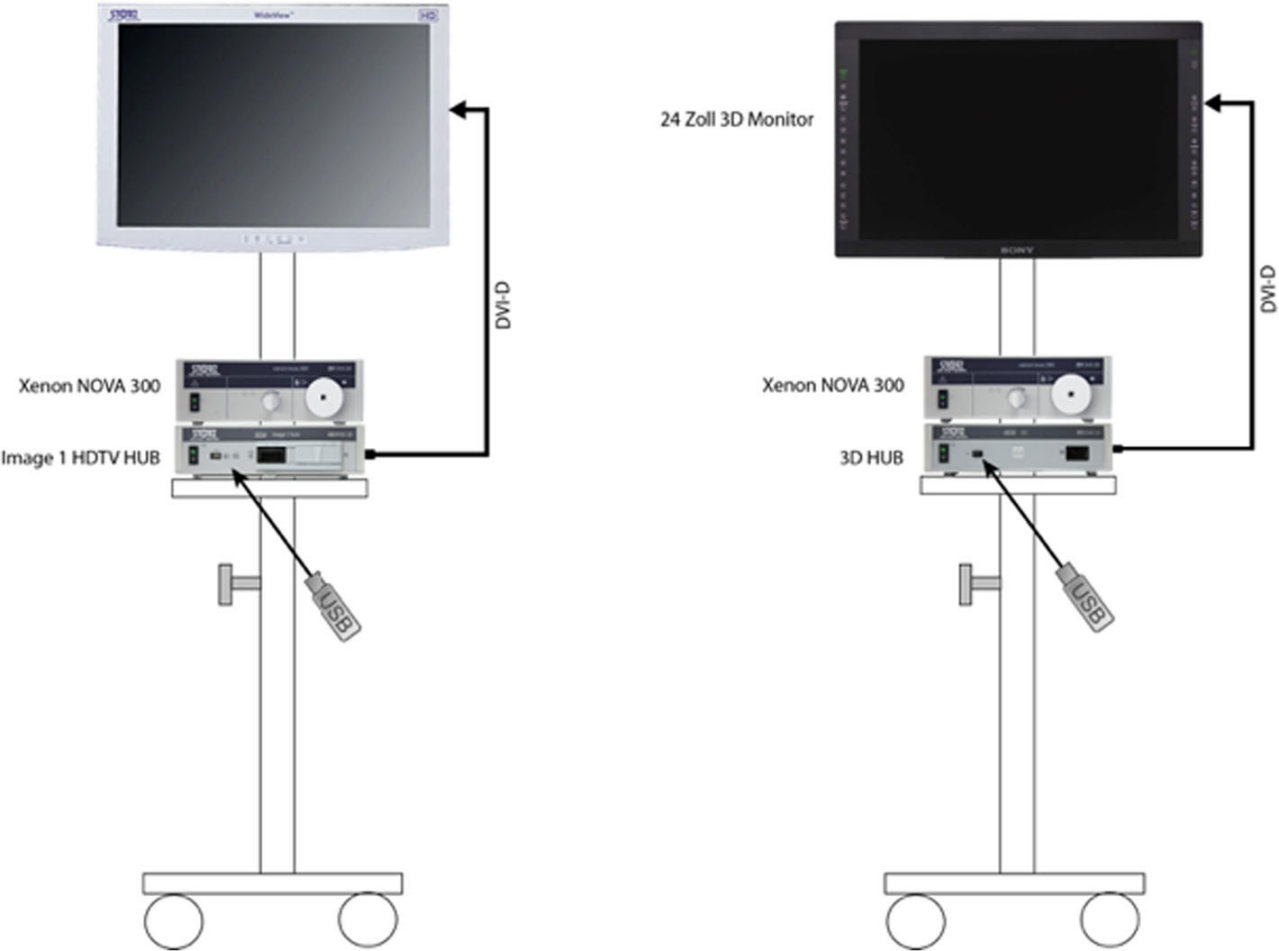
Installation of the 2D and 3D systems

For the 2D system, a Storz Hopkins II, 10 mm, 0° telescope with a Xenon Nova 300 light source and an Image 1 H3-Z Full HD camera (Karl Storz SE & Co., Tuttlingen, Germany) was used. In practice, a 0° optic is not standard anymore, as many surgeons use a 30° optic. At the time this study was conducted, a 3D 30° optic from Storz (Karl Storz SE & Co., Tuttlingen, Germany) was unavailable. Therefore, a 0° optic was used with the 2D system. The 3D system used a Storz 3D TIPCAMR1 with the same Xenon Nova 300 light source (Fig. 2).

### Instructions

The study participants were given a written summary before completing the exercises. All of the details regarding the procedures were explained in short sentences.

Before each task, the participants viewed an introductory video, which included detailed explanations. If participants had any additional questions, they were given the opportunity to ask them prior to beginning the exercises.

### Exercises

Tasks were designed to imitate real surgical scenarios, with the level of difficulty increasing from tasks 1 to 4. In order to measure the amount of time it took for each task to be completed, areas were highlighted to define the initial position of the laparoscopic instruments. Every task started and ended at this position. Errors were recorded and measured using an automatic fault counter for the objective evaluations of tasks 1, 2 and 3. For these purpose the laparoscopic clamps, as well as the area that was off-limits during the exercise, were connected to the counter. However, errors were manually counted for a subjective assessment in tasks 2 and 4. A digital clock with a start/stop feature was used to record the time elapsed at the end of every task and measured the time required for each task to be completed.

**Fig. 3:**
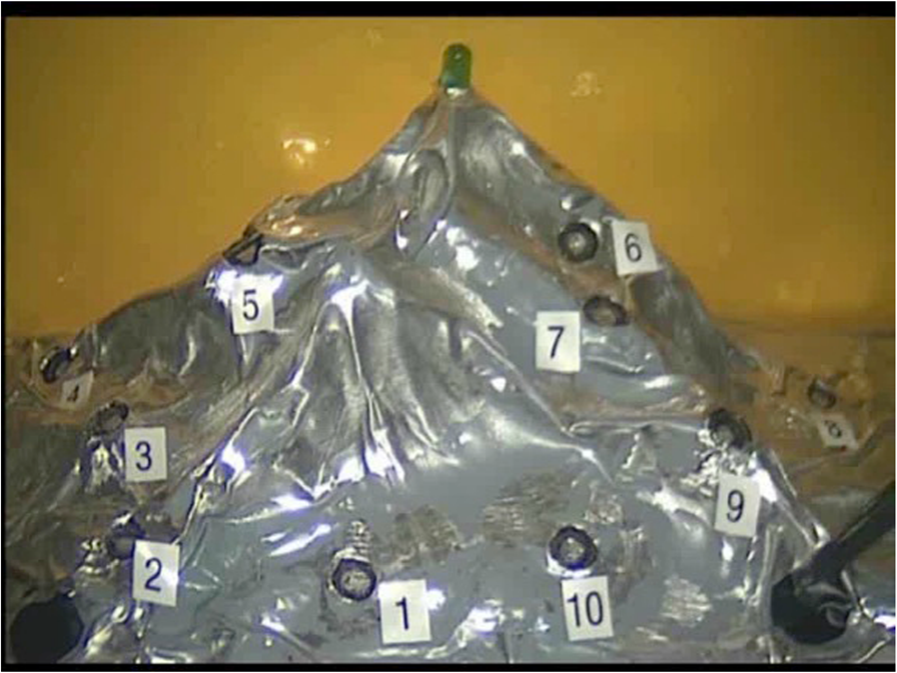
Mountain relief

**Fig. 4:**
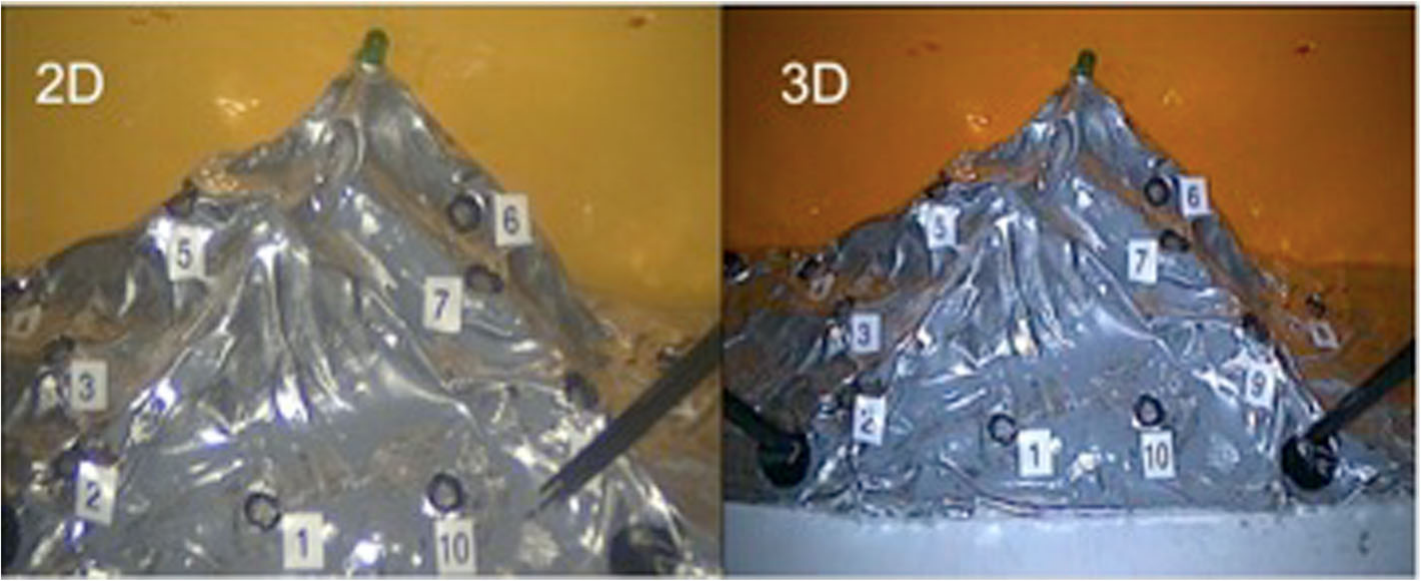
Accuracy

Task 1 *Mountain relief (orientation using 2D and 3D views)* In this task, 10 numbered pins were positioned in a circle (Fig. 3). The goal of the task was to touch only the pins. When a pin was successfully contacted, it produced a light. The task began with the right-hand instrument making contact with pin number 1 and then continued in a clockwise direction. Once completed, the participant repeated the task with their left hand. Contacting the mountain in the wrong area or missing a pin was evaluated as a mistake. The participant was not informed of their mistakes during the task. A comparison between the 2D and 3D views is shown in Fig. 4.

**Fig. 5:**
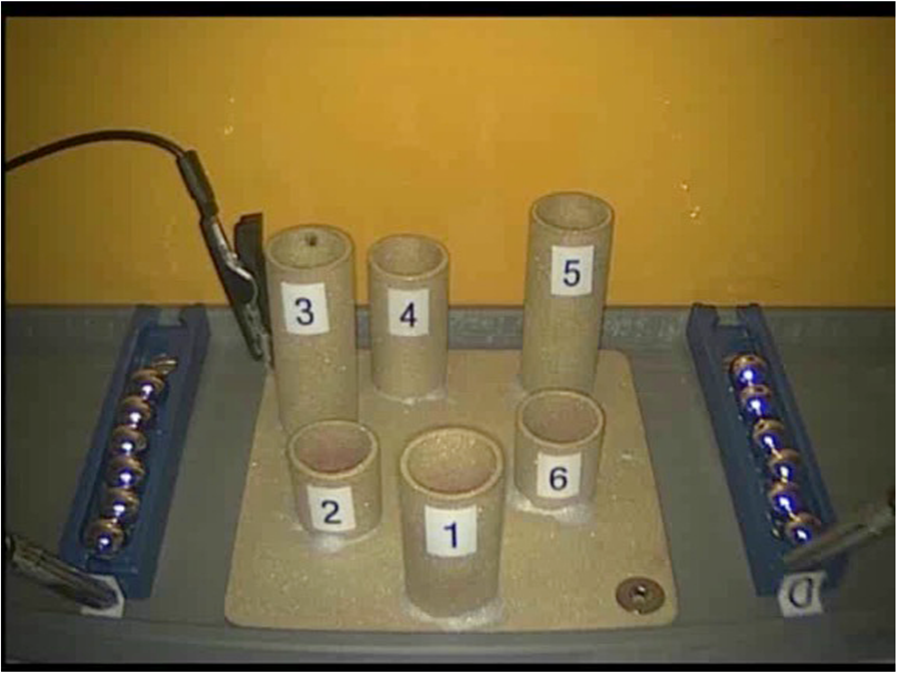
Hot wire

*Task two: Accuracy (Measuring precision in targeting defined points with movements of an object from A to B)* Six empty tubes were placed in a circle on a base plate with 2 ball containers holding 6 balls located on both the right and left sides of the tubes (Fig. 5). The goal of the task was to take the balls from the containers and place them inside the tubes without touching the tubes. After the start signal, the participant took the first ball from a container with the right-hand instrument and loaded tube number 1. They continued with the other tubes in a clockwise direction. Once the right-hand side was completed, the participant completed the same task with the left-hand instrument. If a ball was dropped, it was abandoned and the participant continued with the next one. Touching the tubes or dropping a ball in the wrong tube or on the ground was recorded as a mistake.

**Fig. 6:**
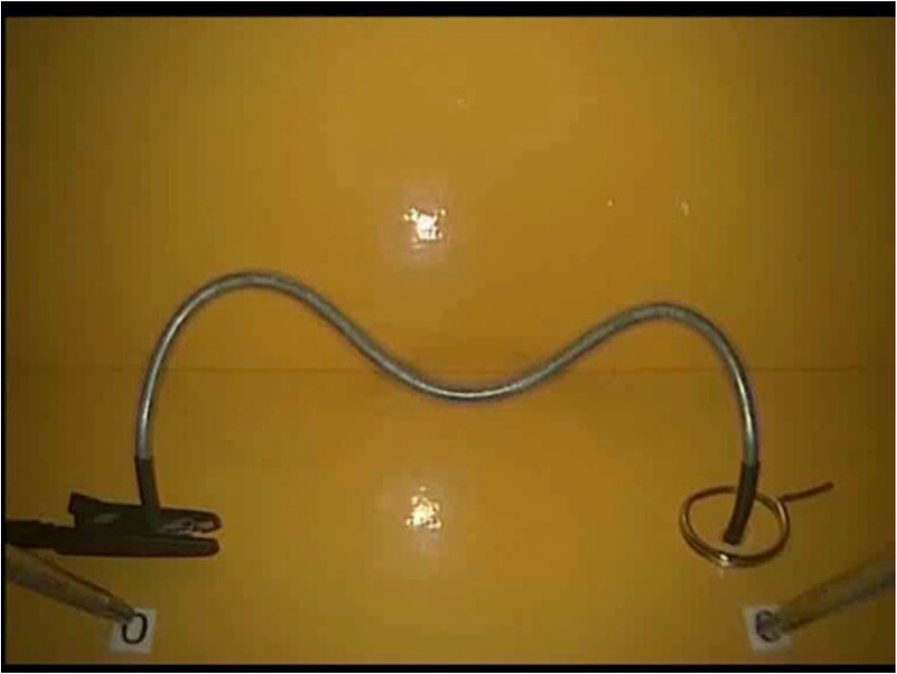
Threading

*Task three: Hot wire (Measuring accuracy, coordination and time for non-linear and continuous movements)* A bent wire, insulated on both ends to rule out mistakes at the start, was attached to a base plate. A ring with an arm was threaded onto the wire (Fig. 6). The purpose of the task was to move the ring along the wire without making any contact. The participant started with the right-hand instrument and then switched to the left-hand one. Touching the wire with the ring was recorded as an error.

**Fig. 7:**
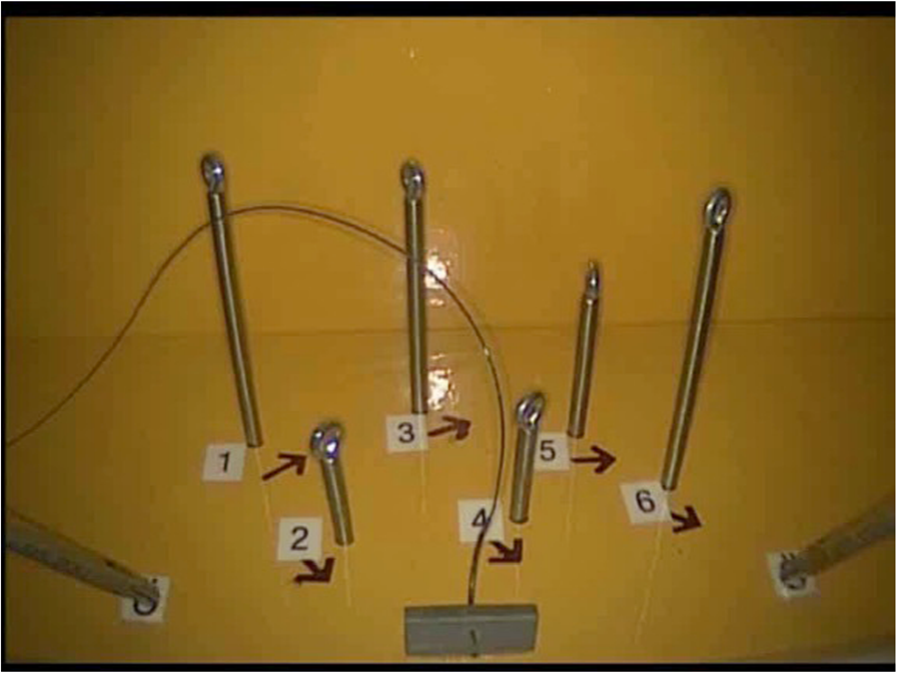
Contrast between 2D and 3D view

*Task four: Threading (Measuring coordination with thread, needle and needle holder)* Six eyelets were fixed to a base plate. All eyelets were numbered and labelled from left to right (Fig. 7). The goal of the task was to thread a V-Loc barbed suture through the first eyelet and then continue to do the same with the others, going from left to right. The participants were allowed to use either their right or left hand to control the needle. Skipping an eyelet, threading in the wrong order or direction, or accidental coiling of the suture were all counted as mistakes.

### Questionnaire

After completing the exercises, participants answered a questionnaire regarding how they felt, both mentally and physically, about their experience using the 2D and 3D systems when completing the tasks.

### Statistical analysis

To prevent selection bias, the order in which participants were given the 2D or 3D system was randomised. An Analysis of Variance with the SYSTAT Statistics software version 13 (Systat Software, San Jose, CA), was used to investigate the difference in means between groups A and B in terms of number of mistakes made and time to complete each of the tasks. Participants experience level, as well as the sequence and the dimensions (3D vs 2D) were controlled for in the analyses. A *P*-value < 0.05 was considered significant.

## Results

### Task 1: Mountain relief

Overall, the participants performed significantly faster (*P* = 0.0001) and made fewer mistakes (*P* = 0.007) with the 3D system. All experience groups spent less time when using the 3D system. The students made fewer mistakes with the system they used in the second round, regardless of the system used in the first round.

The error ratio in the non-expert group was smaller when using the 2D system compared to the 3D one; hence, the type of system had an effect on the number of errors made while performing the tasks. The experts produced fewer mistakes with the system they used first, no matter which system was used (Table 1).

**Table 1:** Summary of task 1 results. Mean value (M) of the used time and mean value (M) of the mistakes made for the 2D and 3D system as well as the corresponding mean difference (MD) between the two systems. Additionally the correlating *P*-values for the difference in time and mistakes of the Group A and B put together

		Group A		Group B	
		Mistakes	Time(s)	Mistakes	Time(s)
Students	M 2D	28.55	146	46.00	173
	M 3D	40.18	127	26.11	99
	MD 2D-3D	−11.64	19	19.89	74
Non-experts	M 2D	34.18	104	32.75	96
	M 3D	36.64	92	33.25	66
	MD 2D-3D	−2.45	12	−0.50	30
Experts	M 2D	24.33	88	23.00	113
	M 3D	23.17	82	24.56	73
	MD 2D-3D	1.17	6	−1.56	40
	*P*-value	Mistakes Group A + B 0.0076		Time Group A + B 0.0001	

### Task 2: accuracy

Participants required significantly less time (*P* = 0.004) for this task, although, there was no significant difference in the mistakes made between groups A and B.

The students and non-experts required less time for the second task no matter which system was used. Experts spent less time when using the 3D system (Table 2).

**Table 2:** Summary of task 2 results. Mean value (M) of the used time and mean value (M) of the mistakes made (a = automatically measured and m = manually measured) for the 2D and 3D system as well as the corresponding mean difference (MD) between the two systems. Additionally the correlating *P*-values for the difference in time and mistakes of the Group A and B put together

		Group A			Group B		
		Mistakes (a)	Mistakes (m)	Time (s)	Mistakes (a)	Mistakes (m)	Time (s)
Student	M 2D	1.91	5.45	137	4.22	6.00	162
	M 3D	3.91	2.27	159	2.89	2.56	135
	MD 2D-3D	−2.00	3.18	−22	1.33	3.44	27
Non-experts	M 2D	4.30	4.82	100	1.00	4.75	90
	M 3D	3.36	2.64	106	3.00	2.50	83
	MD 2D-3D	0.94	2.18	−5	−2.00	2.25	7
Experts	M 2D	10.00	4.17	81	5.88	4.44	101
	M 3D	2.50	2.20	79	2.00	0.88	80
	MD 2D-3D	7.50	1.97	2	3.88	3.57	21
	*P*-value	Mistakes Group A + B 0.7902		Mistakes Group A + B 0.6275		Time Group A + B 0.0041	

### Task 3: hot wire

Performance results for all experience groups showed a significant reduction in time spent completing this task (*P* = 0.0001) and mistakes made (*P* = 0.007).

The students performed the second round more quickly and with fewer mistakes, and the type of system used had no effects. In contrast, the 3D system helped the experts and non-experts reduce their errors (Table 3).

**Table 3:** Summary of task 3 results. Mean value (M) of the used time and mean value (M) of the mistakes made for the 2D and 3D system as well as the corresponding mean difference (MD) between the two systems. Additionally the correlating *P*-values for the difference in time and mistakes of the Group A and B put together

		Group A		Group B	
		Mistakes	Time(s)	Mistakes	Time(s)
Students	M 2D	53.82	151	72.67	205
	M 3D	70.00	207	40.11	149
	MD 2D-3D	−16.18	−56	32.56	56
Non-experts	M 2D	44.18	113	39.75	114
	M 3D	29.55	138	18.00	95
	MD 2D-3D	14.64	−25	21.75	19
Experts	M 2D	40.83	105	44.89	127
	M 3D	25.33	99	34.67	97
	MD 2D-3D	15.50	6	10.22	30
	*P*-value	Mistakes Group A + B 0.0074		Time Group A + B 0.0001	

### Task 4: threading

All experience groups showed a significant decrease in time spent completing this task (P = 0.007) when using the 3D system; although, there were no significant reductions in the number of mistakes made (*P* = 0.488). Group B students took nearly twice as long as Group A with the 2D technique. In the other two experience groups, namely non-experts and experts the time difference was smaller, but still significant (Table 4).

**Table 4:** Summary of task 4 results. Mean value (M) of the used time and mean value (M) of the mistakes made for the 2D and 3D system as well as the corresponding mean difference (MD) between the two systems. Additionally the correlating P-values for the difference in time and mistakes of the Group A and B put together

		Group A		Group B	
		Mistakes	Time(s)	Mistakes	Time(s)
Students	M 2D	0.18	421	0.00	545
	M 3D	0.18	406	0.00	283
	MD 2D-3D	0.00	15	0.00	262
Non-experts	M 2D	0.27	330	0.00	281
	M 3D	0.00	282	0.00	171
	MD 2D-3D	0.27	48	0.00	110
Experts	M 2D	0.00	181	0.00	192
	M 3D	0.00	160	0.00	132
	MD 2D-3D	0.00	21	0.00	60
	*P*-value	Mistakes Group A + B 0.4887		Time Group A + B 0.0073	

### Questionnaire results

**Fig. 8:**
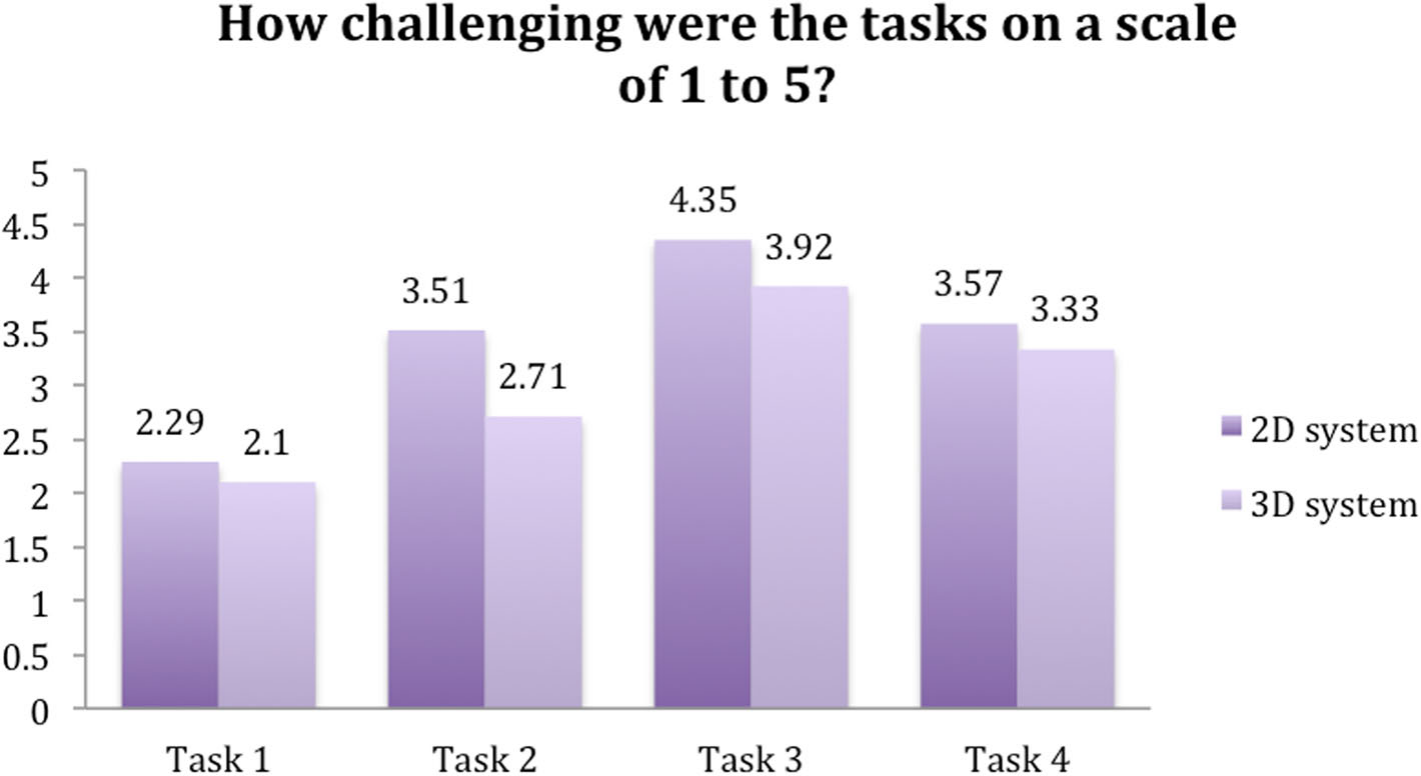
Question 1

**Fig. 9:**
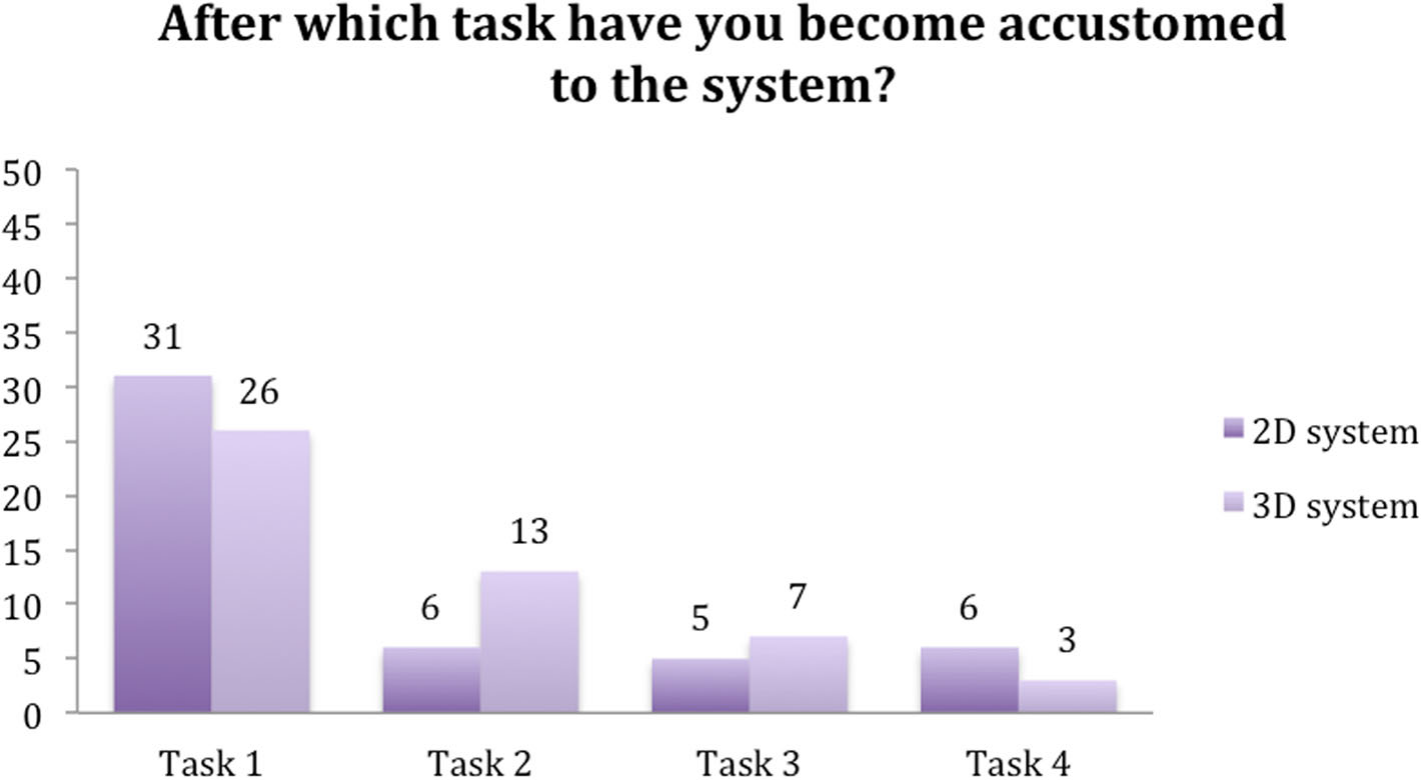
Question 2

Participants rated all of the tasks as more challenging when using the 2D system. Furthermore, participants found that they became accustomed to the 2D system after a single task. In contrast, it took two tasks before subjects were accustomed to the 3D system (Figs. 8 and 9).

**Fig. 10:**
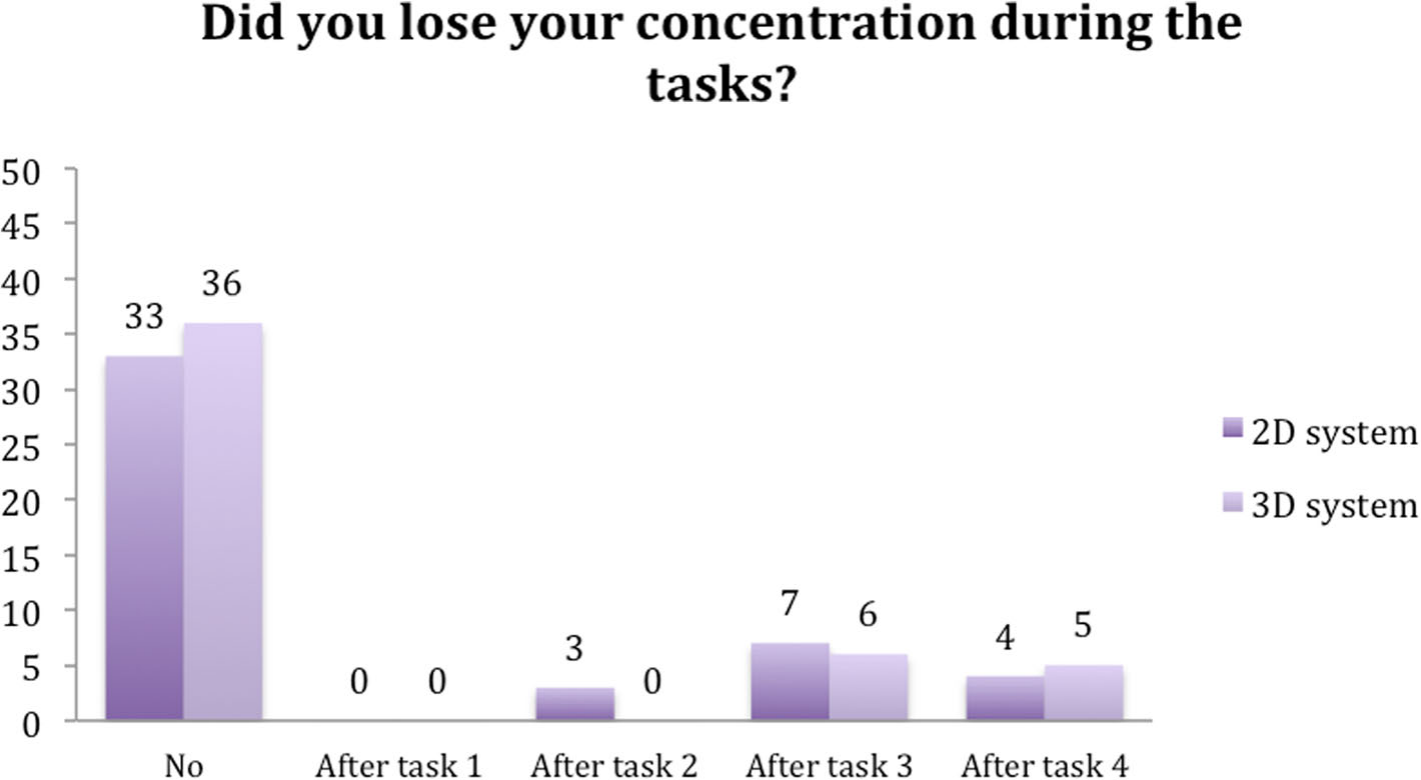
Question 3

**Fig. 11:**
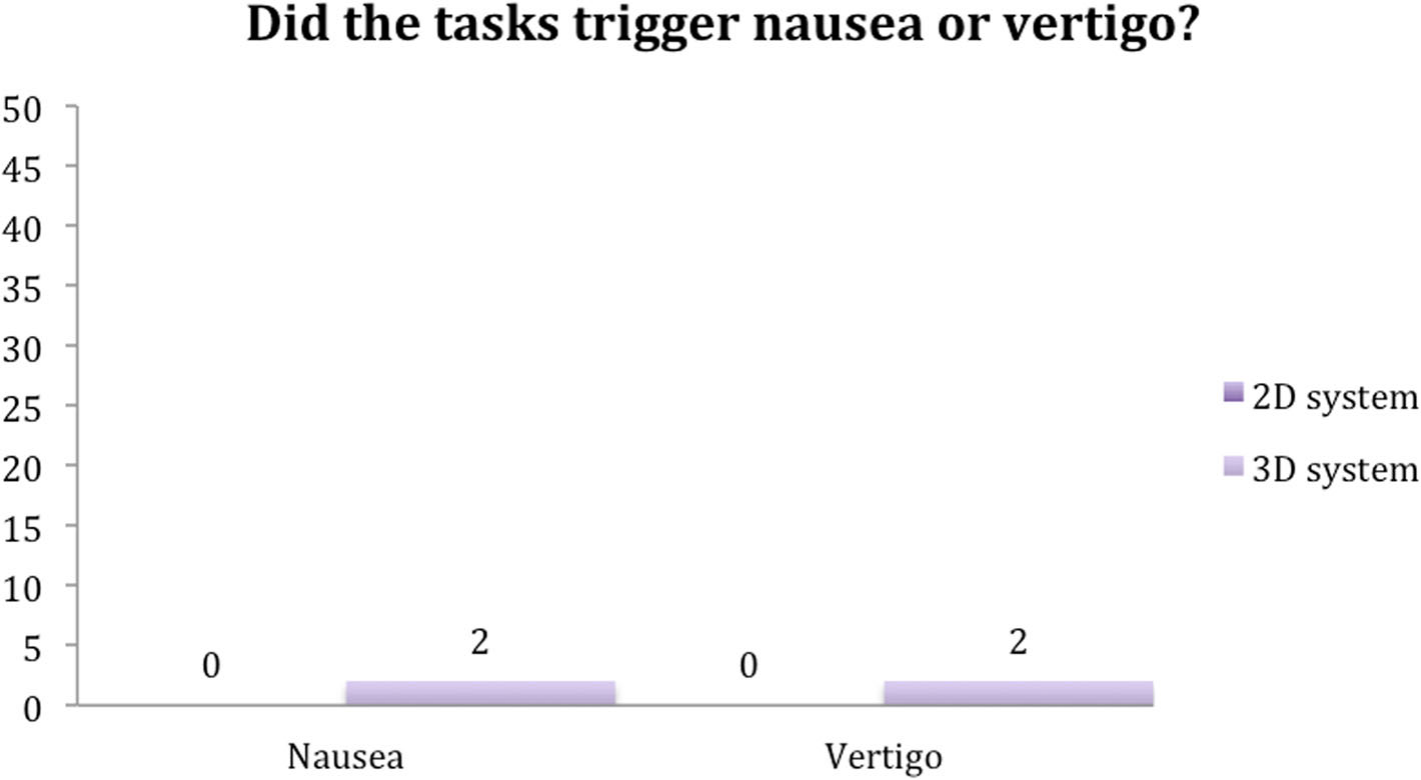
Question 4

Seventy-two percent of the participants reported no lessening of concentration when using the 3D system, while 66% reported no difficulty concentrating when using the 2D system. In addition, only two subjects reported feelings of nausea and dizziness while using the 3D system. There was no nausea or vertigo in 96% of the subjects (Figs. 10 and 11).

**Fig. 12:**
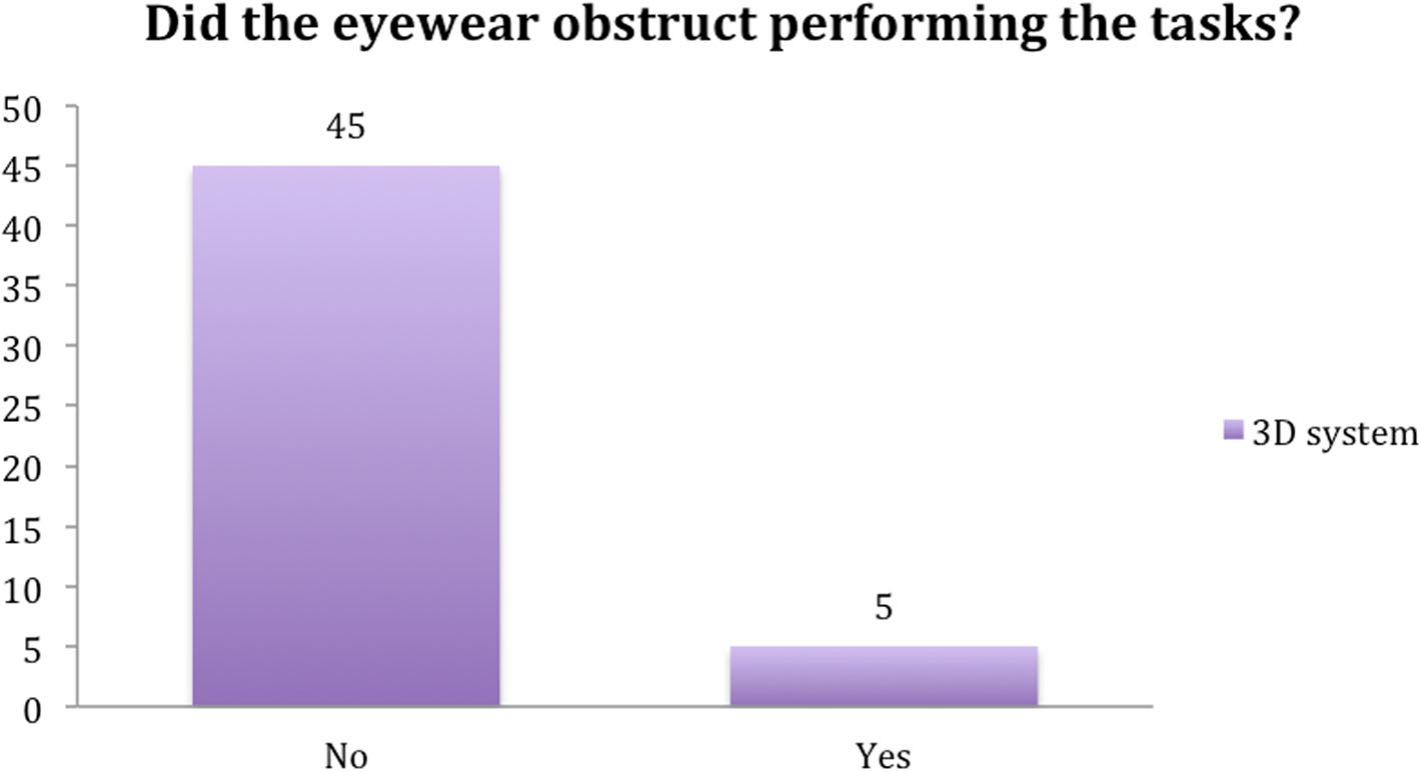
Question 5

**Fig. 13:**
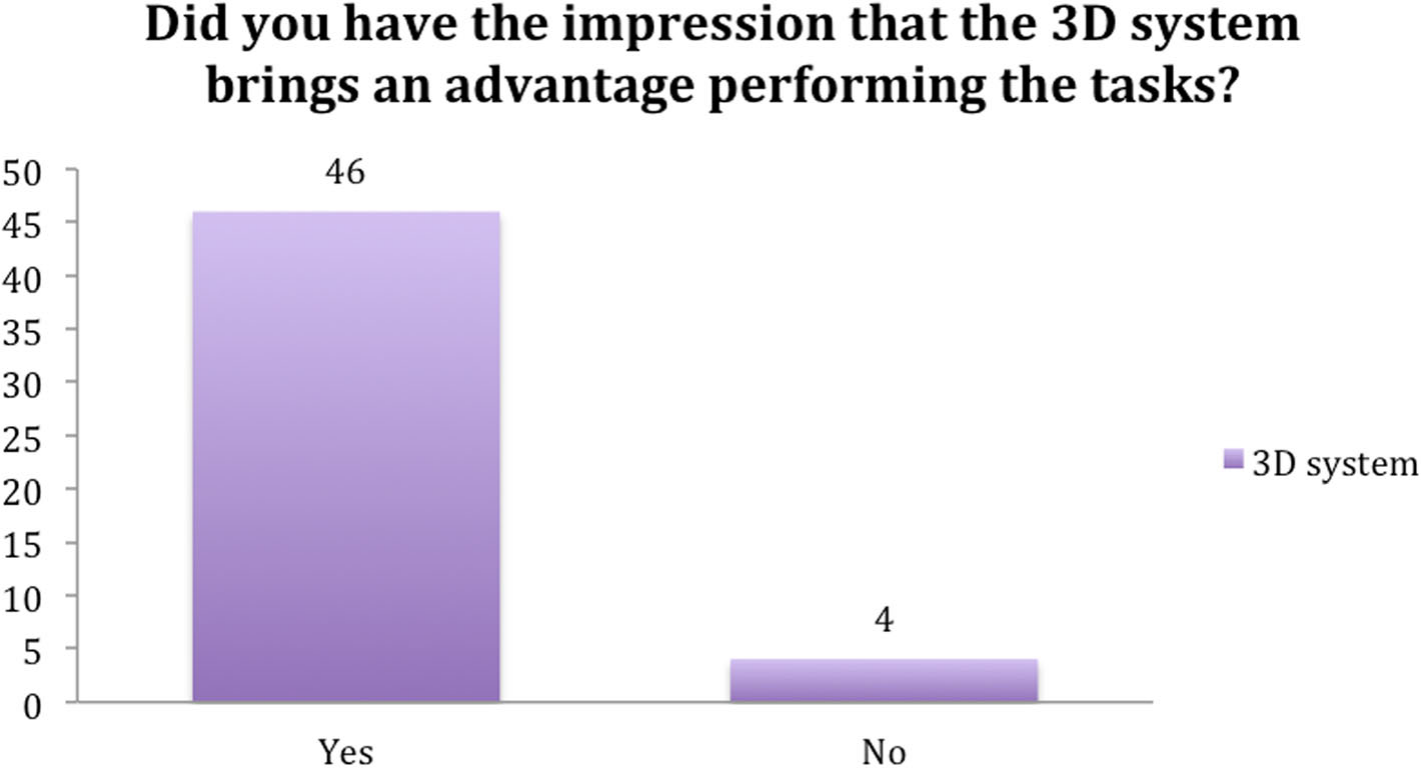
Question 6

When asked if they felt handicapped by the 3D glasses, five participants found the eyewear to be irritating. The reasons given included the glasses fogging-up with movement and losing the 3D view when turning the head sideways. However, more than 90% of subjects considered the 3D system to be more beneficial than the 2D system (Figs. 12 and 13).

## Discussion

The expected advantages of the 3D system include an improved learning curve with greater speed, optimised precision and fewer mistakes that result in shorter operation times, fewer complications and cost reduction.

There was no difference regarding the sequence in which the systems were used, and in all tasks the expert group was significantly faster than the student and non-expert groups.

We anticipated that the 3D system would have a distinct advantage for all of the groups and tasks. Overall, a reduction in mistakes and time was achieved. Group B performed 20% faster, on average, during all 4 tasks and made fewer mistakes in 2 of the tasks compared to group A. The experts significantly benefited from the 3D system in terms of accuracy when performing the ball-throwing exercise compared to the non-experts and students (Table 2). The students, however, showed a significantly greater benefit when using the 3D system in terms of less accidental contact in comparison to experts and non-experts when performing task 3 (Table 3). All participants rated the 3D tasks as easier to perform; and, this rating was significant for the first 3 tasks. The number of tasks that were needed before the candidates adjusted to the perspective did not differ between the 2D and 3D systems. Loss of concentration occurred at the same rate and after the same amount of time for both systems as well. Nausea and dizziness were reported only when working with the 3D system. As a result of the learning effect, the participants performed the second round of trials better than the first round. This effect was especially evident in the beginners, suggestive of a novelty effect, as the more experienced the candidates were, the smaller the effect of the 3D system. According to the subjects’ perceptions, the 3D system gave them an advantage. Nearly 100% of the subjects considered the 3D system as beneficial, with disadvantages, as nausea and dizziness or handicapped by the 3D glasses because of the glasses fogging-up with movement and losing the 3D view when turning the head sideways, noticed only occasionally.

This work supports previous studies investigating the benefits of this technology and explains the disadvantages in detail, such as the hindrance experienced when using goggles, loss of concentration and headache [7–10]. Measurements were also taken from non-experts. Doctors-in-training have not previously participated in research measuring the benefits of 3D. Their inclusion is another factor that suggests the 3D technique can be easily incorporated into routine LSCs, which are currently an important aspect of modern surgery [11–13]. There are examples of robot-assisted LSCs, such as the Da Vinci, which benefits from 3D visualization and increased degrees of freedom to provide better results [14, 15]. However, the combination of 3D and conventional LSC offers a more cost-effective and simpler alternative to the Da Vinci [16, 17]. Yet, this technology is still in the developmental phase, thus our work should be seen as a contribution to help move this technology forward [11, 18–20].

However, there is room for improvements in future projects. The major limitation of this study is that a 0° optic was used with the 2D system as a 3D 30° optic from Storz (Karl Storz SE & Co., Tuttlingen, Germany) was unavailable at the time this study was conducted. Using a 0° optic is not standard anymore, thus, some of the experts or non-experts may have experienced issues with the unusual perspective and this could have affected the performance with the 2D system. Then again, all the participants performed the exercises under the same conditions. The second limitation of this study is that the number of participants is small. This is partly the result of the strict inclusion criteria for the experts and then again due to the difficulty of recruiting non-experts. Though, compared to other comparative studies exploring the role of the 3D and 2D system in laparoscopy the recruited number of participants is similar [21–23]. The third limitation of the current study is the challenging interpretation of the results on the basis of the inhomogeneous composition of the two groups as a result of the conducted randomization. The candidates were randomized in order to prevent a selection bias. The initial randomization proved to be correct. There was no difference regarding the sequence in which the systems were used and no difference of the performance could be detected if you look at the same experience level in the respective groups. However, a crossover study without this type of randomization could be more suitable for a comparative pelvitrainer study [22–24].

In summary this study suggests, surgeons should start using 3D systems early in their operating career, as the effects are substantial for beginners and the learning curve can be improved. This is especially important as there are fewer operations due to an increase in the number of conservative procedures based on better alternative treatments or diagnostics [25–30]. In some clinics, the 3D system is available in the operation theatre as a permanent feature, but is rarely used. This could be also an approach that may prove useful in increasing the use of robot-assisted LSC using simple tools. A multi-centre study comparing the outcome of operations using 3D systems in conventional LSC and robot-assisted LSC should be considered for future studies.

## Conclusion

Irrespective of experience level, 3D laparoscopy shows advantages in saving time, increasing accuracy and reducing mistakes. These benefits were also accompanied by subjective advantages that were noted by the participants. However, the more complex the task, the less significant the benefit of the 3D system and some people feel handicapped by the eyewear.

## Data Availability

The datasets analysed during the current study are available from the corresponding author on reasonable request.

## Abbreviations

3D: 3 dimensional
2D: 2 dimensional
LSC: Laparoscopy
IRB: Institutional review board
HD: High definition
